# Novel methods for the rapid detection of trace tetracyclines based on the fluorescence behaviours of Maillard reaction fluorescent nanoparticles

**DOI:** 10.1039/d0ra05298a

**Published:** 2020-12-08

**Authors:** Xue-jing Si, Hong-ling Wang, Tun-hua Wu, Ping Wang

**Affiliations:** School of Pharmaceutical Sciences, Wenzhou Medical University Wenzhou 325035 China wangpingxmu@163.com +86-577-86689745 +86-577-86689949; School of Information Engineering, Wenzhou Business College Wenzhou 325035 China

## Abstract

The Maillard reaction and its fluorescent products have attracted widespread attention in the field of food safety and biology. Herein, the novel Maillard reaction fluorescent nanoparticles (MRFNs) as a fluorescent probe were synthesized *via* a “green” method with simple technical processes. In addition, the effects of tetracycline (TC) and chlorotetracycline (CTC) representing certain properties of tetracyclines (TCs) on the fluorescence behaviour of MRFNs were studied, respectively. The present study showed that the fluorescence intensity of MRFNs greatly enhanced with a linear increase in the CTC concentration. However, with the gradual increase in the TC concentration, the intensity of MRFNs tended to significantly decrease linearly. Based on this, novel fluorescence analysis methods for the simple and rapid detection of TC and CTC in water bodies were established, respectively. Significantly, the proposed detection methods were successfully adopted for detecting TC and CTC in some environmental water samples. Besides, the possible mechanisms for TC-induced fluorescence quenching and CTC-induced fluorescence enhancement of MRFNs were also discussed, respectively.

## Abstract 2

The Maillard reaction and its fluorescent products have attracted widespread attention in the field of food safety and biology.

## Graphical abstract

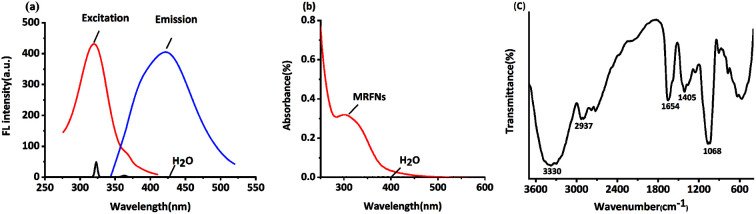

## 1. Introduction

Antibiotics play important roles in human therapeutic medicine and have caused a huge revolution since their discovery in 1929. They have been widely used in the treatment of human diseases and the growth of animals and plants owing to their antibacterial and anti-inflammatory effects.^1^ However, due to the release of antibiotics into the environment that causes antibiotic pollution, the hazards combined with antibiotic contamination affect people to a large extent.^2,3^ In addition, the determination of antibiotics in ground water and effluents has always been a challenge because of the interference that might occur during the analysis due to the complexity of the matrix that dissolves the solute, and the time needed to perform analysis and produce a reliable result.^4–7^ Tetracyclines (TCs) are a class of extremely common antibacterial broad-spectrum antibiotics that are diffusely used in human and animal communities.^8,9^ However, because of their extensive use, TC residuals cause potential harm such as genetic changes, mutations and other changes in the human microbiota, resulting in bacterial resistance.^10,11^ Therefore, it is necessary to establish simple, rapid, and economic methods to detect TCs in aqueous solutions.

Basically, to quantify TCs in water, analytical chemists go through standardized procedures starting by the sample preparation step that includes concentration and cleaning processes towards analysis.^12,13^ Classical analytical techniques used to monitor such chemicals including high performance liquid chromatography (HPLC) equipped with various sensitive detectors, monitor such chemicals that could be placed in series of so-called hyphenated techniques, mass spectrometry (MS) and gas chromatography (GC) also equipped with appropriate detectors like the electron capture detector (ECD), the flame ionization detector (FID), and the MS detectors of different mass analyzers' configuration.^14–16^ Most of these techniques are first based on the separation process allowing analytes to reach the detector according to their retention time; however, these separation and extraction procedures are complicated and might consume large amounts of organic solvents. Relatively speaking, fluorimetry could have the advantages of easy operation, low cost, and high sensitivity in performing the assay of trace fluorophores. However, owing to the generally low fluorescence quantum yield of TCs, fluorimetry is limited in TC research. In recent years, researchers have been exploring numerous fluorescent probes to analyze and detect TCs.^17–20^

The Maillard reaction is called the “non-enzymatic browning reaction”, and its products are coloured substances produced by the reaction of sugar (pentose and hexose) with amino acids, dipeptides or tripeptides, which are widely used in bacteriostasis, antioxidation and improvement of food colour and flavour.^21,22^ In recent years, it has been investigated that some Maillard reaction products made from specific raw materials can be used to determine some ions or small molecules.^23,24^ However, all methods for preparing the Maillard reaction products above are hydrothermal methods, which require a large amount of time. The preparation of the Maillard reaction products *via* a microwave heating method can greatly save the preparation time and simplify the synthesis process.^25^ So far, there is no method for detecting TCs in aqueous waters utilizing Maillard reaction products obtained by the microwave heating method as fluorescent probes.

In this study, Maillard reaction fluorescence nanoparticles (MRFNs) were synthesized by “green” methods with simple technical processes and low-toxicity compounds. In addition, chlorotetracycline (CTC) and tetracycline (TC), which represent certain properties of TCs, were chosen as test subjects. Then, the effects of TC and CTC on the fluorescence behaviours of MRFNs were inspected, respectively. Based on this, new fluorescence analysis methods for the rapid detection of TC and CTC in aqueous solutions were established, respectively. Finally, the proposed methods were adopted for detecting TC and CTC in several environmental water samples, and the related mechanisms for TC-induced fluorescence quenching and CTC-induced fluorescence enhancement have been discussed.

## 2. Experimental sections

### 2.1 Instruments and reagents

UV-visible absorption spectra and fluorescence spectra were recorded on a UV-2550 spectrophotometer (Shimadzu, Japan) and an RF-5301 fluorescence spectrophotometer (Hitachi Corporation, Japan), respectively. The fluorescence lifetime was measured by an FS5 fluorescence spectrometer (Edinburgh Instruments, England). The infrared spectra were recorded by an FTIR-850 Fourier transform infrared spectrometer. The hydrodynamic diameter of MRFNs was analysed by a Litesizer 500 laser particle size analyser (Anton Paar, Austria). The sample was lyophilized with an Alpha 2-4 LD plus freeze dryer (Christ, Germany). Microwave heating was performed using a G8023CSL-K3 microwave oven (Galanz, China).

TC (C_22_H_24_N_2_O_8_·HCl, purity >97%) and CTC (C_22_H_23_ClN_2_O_8_·HCl, purity >94%) were purchased from Dr. Ehrenstorfer GmbH (Germany). They were dissolved in ultrapure water and appropriately diluted the solutions to prepare working solutions. d-Glucose (C_6_H_12_O_6_·H_2_O, Xilong Scientific Co., Ltd.), l-arginine (C_6_H_14_N_4_O_2_, Shanghai Lanji Technology Development Co., Ltd.), citric acid (C_6_H_8_O_7_·H_2_O, Chinese Pharmaceutical Group Chemical Reagent Co., Ltd.), disodium hydrogen phosphate (Na_2_HPO_4_·12H_2_O, Xilong Scientific Co., Ltd.) and nine metal ions (Cu^2+^, Li^+^, K^+^, Cs^+^, Zn^2+^, Ni^2+^, Na^+^, Co^2+^, and Mg^2+^) were also used in this experiment. All reagents were of analytical pure grade and did not require further purification before use.

### 2.2 Preparation of MRFNs

In brief, d-glucose (0.20 g) and l-arginine (0.03 g) were dissolved in ultrapure water and treated *via* ultrasonication for approximately 3 min to disperse them evenly. Then, the mixture was transferred to a microwave oven. The power was set to 480 W, and the reaction time was 7 min. After the reaction was completed, the mixture was sufficiently cooled and filtered through a 0.22 μm membrane. The size of the obtained MRFNs was detected by a Litesizer 500 laser particle size analyser.

### 2.3 The detection procedures of TC and CTC in aqueous solutions

First, a series of TC working solutions (80 μg mL^−1^) with different volumes (0, 20, 40, 100, 200, 400, 600, 800, 1000, 1200, 1400, 1600, 1800, and 2000 μL) were thoroughly mixed with the MRFNs (500 μL). A citrate-phosphate buffer solution (CPBS, pH = 7) was added to mixtures. Make up to 5 mL with ultrapure water. Then, all solutions were mixed and incubated fully for 5 min at room temperature. Finally, the fluorescence spectra were determined.

The detection procedures for CTC were basically the same as discussed above. A series of CTC working solutions (60 μg mL^−1^) with different volumes (0, 10, 30, 50, 100, 200, 300, 400, 500, and 600 μL) were thoroughly mixed with MRFNs (180 μL). The CPBS buffer solution was also added to each of the mixtures. Finally, the fluorescence analysis was separately performed.

## 3. Results and discussion

### 3.1 Spectral properties of MRFNs in aqueous solutions

**Fig. 1:**
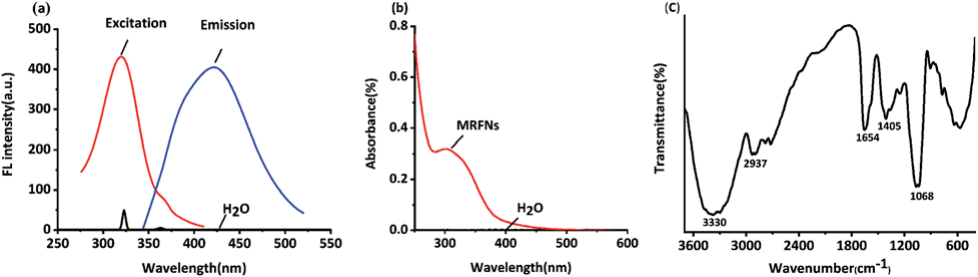
(a) Fluorescence excitation and fluorescence emission spectra of MRFNs, (b) UV-visible absorption spectra of MRFNs, and (c) infrared spectra of MRFNs.

In this study, the fluorescence spectra of MRFNs were studied. As shown in Fig. 1a, MRFNs displayed intense intrinsic fluorescence in aqueous solutions. It can be excited at 323 nm and emitted at 425 nm. In addition, it can be seen from the spectra that the fluorescence signal of the solvent would not interfere with the detection of MRFNs in aqueous solutions. As shown in Fig. 1b, MRFNs had a distinct UV absorption peak at approximately 310 nm, which might be ascribed to the n–π* transition of the C=O bond.^26,27^ The experiment results of the infrared spectroscopy about the MRFNs are shown below: there is a strong and wide peak at 3330 cm^−1^, which might be attributed to the structure of hydroxyl groups. Moreover, the characteristic peak of alcohol near 1068 cm^−1^ could further confirm the existence of hydroxyl groups. The peak at 2937 cm^−1^ can be attributed to the stretching vibration of –CH_2_–, and the peak at 1654 cm^−1^ can be attributed to the stretching vibration of C=N. The peak at 1405 cm^−1^ is attributed to the bond of –COO– (Fig. 1c). The existence of these functional groups was the basis for the stable existence of MRFNs in aqueous solutions.

### 3.2 Effects of TC and CTC on the fluorescence behaviours of MRFNs in aqueous solutions

**Fig. 2:**
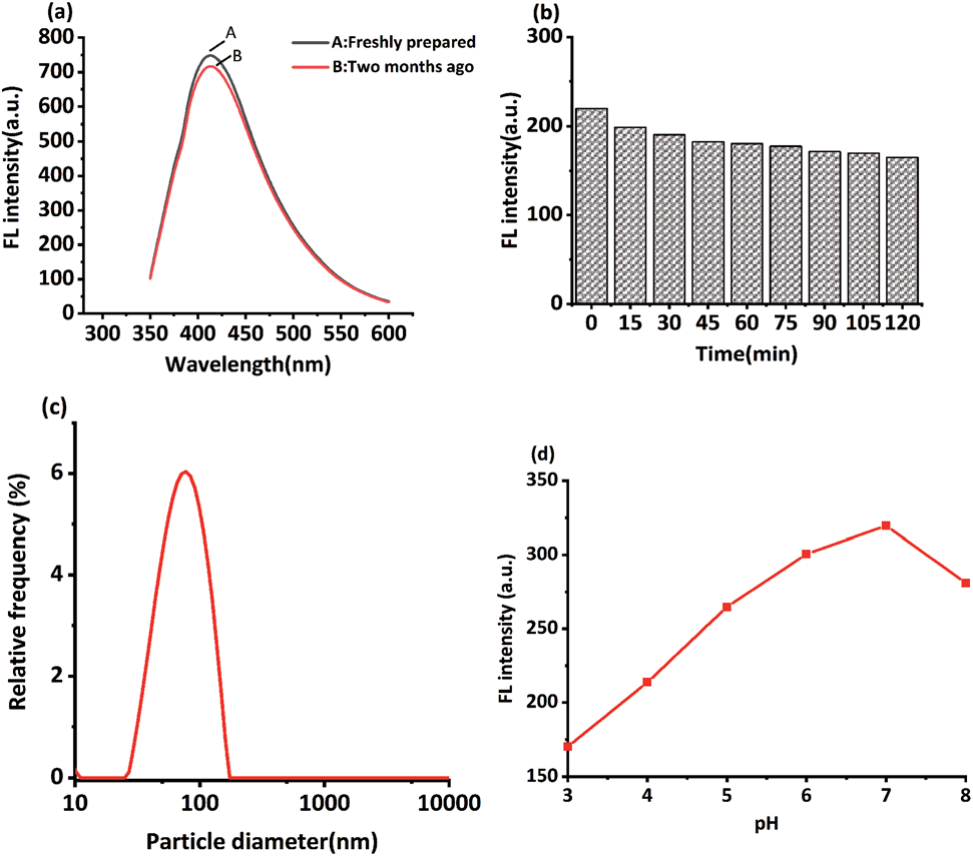
(a) The fluorescence intensity of MRFNs before and after two months, (b) the fluorescence intensity of MRFNs under ultraviolet radiation (365 nm), (c) particle diameter of MRFNs and (d) the influence of pH on the fluorescence intensity of MRFNs.

**Scheme 1:**
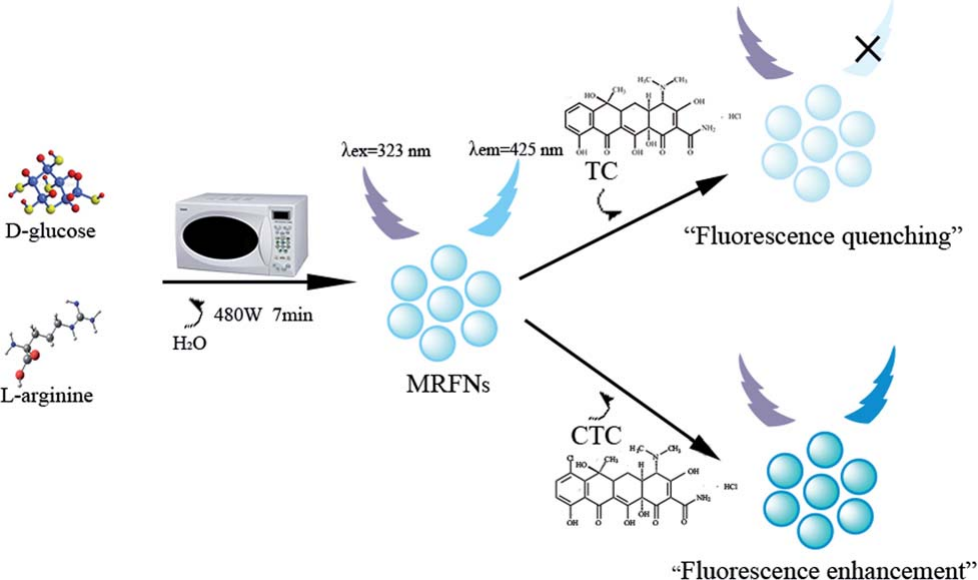
Schematic of the synthesis of MRFNs and the fluorescent sensing process for TC and CTC in aqueous solutions.

**Fig. 3:**
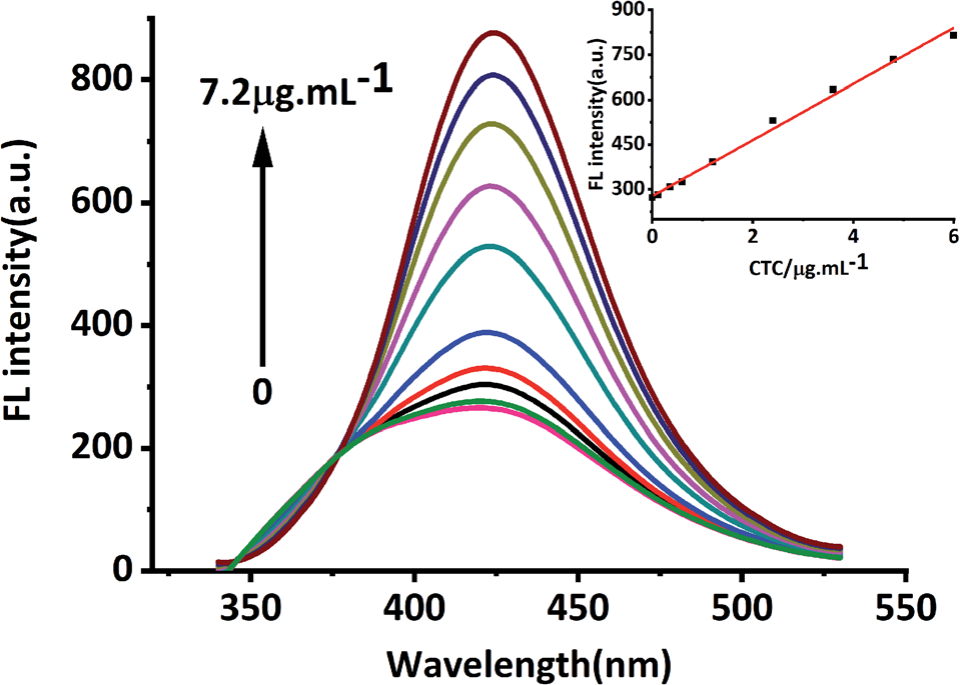
Fluorescence response of MRFNs at different concentrations of CTC (inset shows the linear relationship between fluorescence intensity and various CTC concentrations).

**Fig. 4:**
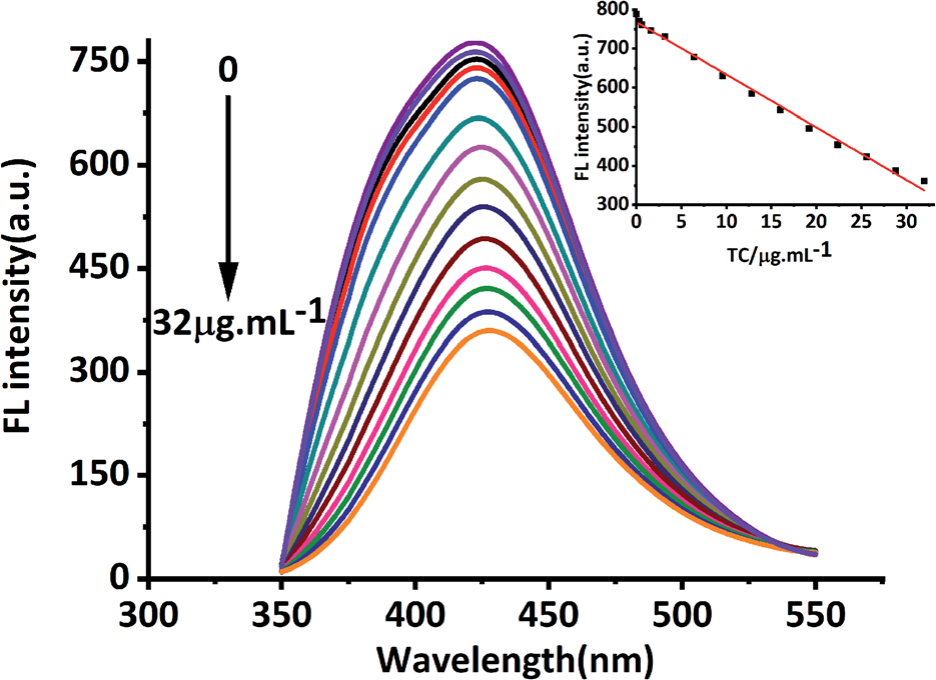
Fluorescence response of MRFNs at different concentrations of TC (inset shows the linear relationship between fluorescence intensity and various TC concentrations).

In this study, MRFNs in a canary yellow solution were synthesized using the “green” method with simple technical processes and low-toxicity compounds. The stability of the as-prepared MRFNs was tested, and Fig. 2a and b show that MRFNs had good stability and anti-photobleaching property. The intensity-weighted analysis results displayed that the average particle diameter of MRFNs in aqueous solutions was 72 ± 1.0 nm (Fig. 2c). As shown in Fig. 2d, the effect of pH on the fluorescence intensity of MRFNs was investigated, and it was found that under the condition of pH 7, MRFNs showed the strongest fluorescence emission; therefore, pH 7 was selected as the controlled pH in all detection processes. It was well known that TC and CTC had very similar chemical structures and similar ultraviolet absorption spectra. In order to further study the effects of each TC on the fluorescence behaviours of MRFNs, the fluorescence intensities of MRFNs mixed with each TC were investigated, respectively. As illustrated in Scheme 1, under identical experimental conditions, CTC and TC had opposite effects on the fluorescence behaviours of MRFNs. The results showed that the fluorescence intensity of MRFNs greatly enhanced with the increase in the concentration of CTC (Fig. 3), and with the gradual increase in the TC concentration, the fluorescence intensity of the MRFNs tended to significantly decrease (Fig. 4).

**Fig. 5:**
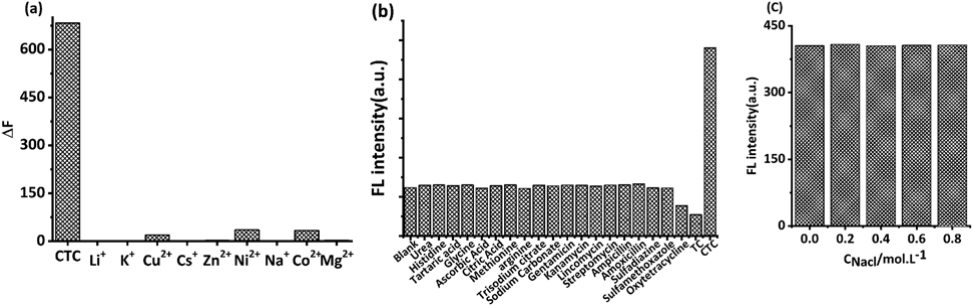
(a) Fluorescence changes (Δ*F*) of MRFNs caused by CTC (4.8 mg L^−1^) and nine ions (Li ^+^, K^+^, Cu^2+^, Cs^+^, Zn^2+^, Ni^2+^, Na^+^, Co^2+^, Mg^2+^, 40 mg L^−1^), (b) the interference of some common small molecules and other kinds of antibiotics (CTC: 10 mg L^−1^, other substances: 32 mg L^−1^), and (c) effects of NaCl on the fluorescence intensity of MRFNs.

Previous studies reported that the Maillard products prepared from some raw materials had strong ionic complexing ability.^24^ In this study, the influence of nine types of ions on MRFNs was investigated using CTC as a reference. As shown in Fig. 5a, the changes in the MRFN fluorescence caused by CTC were significantly greater than that caused by each cation, whose concentrations were nearly ten times higher than CTC, indicating that this method has good anti-ion interference capability. In addition, the effects of some common small molecule substances and other kinds of antibiotics on the fluorescence intensity of MRFNs were investigated, and the results showed that MRFNs have good selectivity towards TCs (Fig. 5b). Moreover, the effect of NaCl on the fluorescence intensity of MRFNs was also investigated. It can be seen from Fig. 5c that the fluorescence intensity of MRFNs was basically stable in the concentration range of 0 to 0.8 mol L^−1^, which showed the excellent salt resistance of MRFNs in aqueous solutions. It is well known that the concentration of saline fluid in humans is approximately 0.15 mol L^−1^. Therefore, the results discussed above might apply to study various kinds of body fluids that are rich in physiological salts.

### 3.3 Establishment of methods for detecting TC and CTC in aqueous solutions

From the studies above, the fluorescence behaviours of MRFNs influenced by TC and CTC were observed in aqueous solutions. The fluorescence intensities of MRFNs showed exactly the opposite changes in the presence of TC and CTC, and different concentrations of TCs had different degrees of influence on the fluorescence intensity of MRFNs. Moreover, the fluorescence intensity of MRFNs showed a linear downward trend with the increase in the TC concentration within a certain range, and with the increase in the CTC concentration, the fluorescence intensity of MRFNs showed a linear increasing trend (Fig. 2 and 3). Therefore, novel fluorometric methods for detecting TC and CTC in aqueous solutions were successfully established, respectively. Table 1 lists the calibration equation, linear range, correlation coefficient and detection limit (LOD, 3*δ*/*k*) of the established methods. Furthermore, due to the diversity of fluorescent probes used to detect TCs, some analytical methods using probes to detect CTC and TC in recent years are summarized and compared with the established methods above (Table 2).

**Table 1:** Performance data for detecting TC and CTC in aqueous solutions

Analyte	Linear range (μg mL^−1^)	Calibration equation	Correlation coefficient	LOD (ng mL^−1^)
TC	0.32–32	*y* b = −13.471*x*^a^ + 767.98	0.9942	278.0
CTC	0.12–6.0	*y* = 93.564*x* + 278.160	0.9936	40.0

a Concentration of each TC (μg mL^−1^).

b Relative fluorescence intensity of each TC.

**Table 2:** Comparison of different analytical methods for detecting CTC and TC with fluorescent probes

Type of TCs	Fluorescent probe	Function mode	Linear range (μM)	LOD (μM)	Reference
CTC	N-CQDs	Fluorescence quenching	—	0.279	19
	l-Tryptophan	Fluorescence quenching	0.65–30	0.2	28
	CDs	Fluorescence quenching	1–300	0.36	27
	N-CDs	Fluorescence quenching	≈1.78–42.56	≈0.163	29
	MRFNs	Fluorescence enhancement	≈0.23–1.16	≈0.076	This work
TC	QDs	Fluorescence quenching	15–600	7.780	30
	N-CQDs	Fluorescence quenching	3.32–32.26	0.237	19
	Nanodots	Fluorescence quenching	2–150	0.52	31
	MoS_2_ QDs	Fluorescence quenching	—	6.52	32
	Carbon dots	Fluorescence quenching	10–400	6	33
	N-CDs	Fluorescence quenching	2–200	0.6	17
	MRFNs	Fluorescence quenching	≈0.67–67	≈0.579	This work

### 3.4 Detection of TC and CTC in environmental water samples

To evaluate the analytical characteristics of the proposed methods, the methods were utilized to detect TC and CTC in environmental water samples (tap water, mineral water and lake water). The three types of water samples were treated only by simple filtration to remove the insoluble substrates. To be consistent, the selected concentrations of the TC and CTC solutions were added to each of the three water samples, the measurements were repeated three times, respectively. The results indicated that the recovery of each TC remained within the range from 92% to 108% for the three types of environmental water samples, which was basically satisfactory (Table 3). Therefore, the established methods were reliable and appropriate for the detection of TC and CTC in three types of environmental water samples.

**Table 3:** Data for detecting TC and CTC in three environmental water samples^a^

Water sample	Internal standard substances (μg mL^−1^)	Detection quantity (μg mL^−1^)	Recovery (%)	Relative standard deviation
1	16.0	16.17	101.06%	0.0036
2	16.0	16.32	102.00%	0.0221
3	16.0	14.86	92.88%	0.0023
4	3.0	2.96	98.72%	0.0063
5	3.0	3.11	103.67%	0.0141
6	3.0	3.26	108.67%	0.0001

a (1) TC in tap water, (2) TC in mineral water, (3) TC in lake water, (4) CTC in tap water, (5) CTC in mineral water, (6) CTC in lake water.

### 3.5 Possible mechanisms for TC-induced fluorescence quenching and CTC-induced fluorescence enhancement of MRFNs

**Fig. 6:**
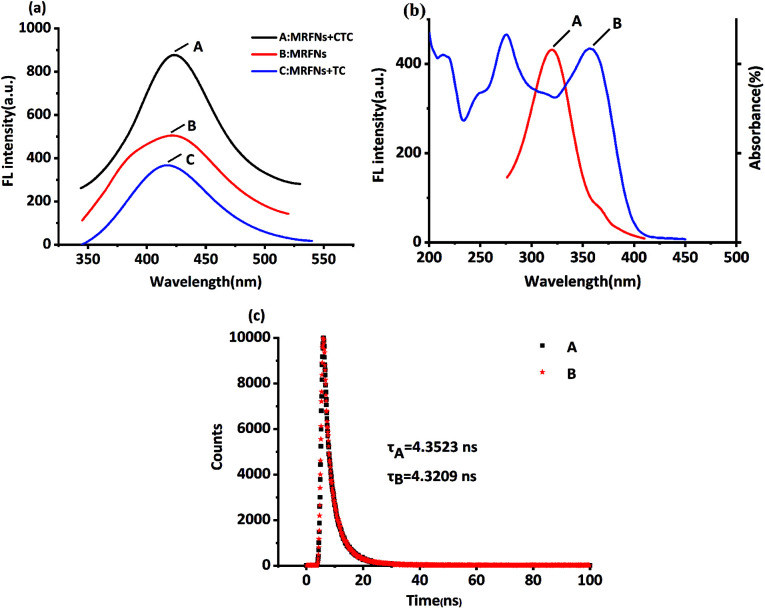
(a) Fluorescence spectra of MRFNs in the presence of CTC (A), MRFNs (B) and MRFNs in the presence of TC (C), (b) excitation band (A) of MRFNs and ultraviolet absorption band (B) of TC, and (c) fluorescence lifetime of MRFNs in the absence (A) and presence (B) of TC.

It was well known that the chemical structure of CTC had only one more chlorine atom compared to that of TC. From the results above, TC and CTC had opposite effects on the fluorescence behaviours of MRFNs in aqueous solutions. Also, the results revealed that the shapes and locations of the maximum fluorescence peak of MRFNs were only slightly different after adding each of TCs (Fig. 6a). In addition, the experimental results also demonstrated that the TC absorption band overlapped with the excitation band of MRFNs (Fig. 6b). Given these, the fluorescence quenching effect of TC on MRFNs was speculated about the internal filtration effect (IFE) or fluorescence resonance energy transfer (FRET). The fluorescence lifetime of MRFNs before and after adding TC was further measured. As shown in Fig. 6c, the fluorescence lifetime of MRFNs was 4.3523 ns, and the mixture after adding TC was 4.3209 ns. The essentially constant fluorescence lifetime confirmed that the quenching process belonged to static quenching. Many studies showed that FRET would significantly change the fluorescence lifetime due to energy transfer.^33,34^ Therefore, an efficient IFE might be the possible mechanism for the fluorescence quenching of MRFNs caused by TC.

Few studies have investigated the mechanisms of the fluorescence enhancement by TCs. Moreover, due to the limitation of experimental conditions, the reason why CTC may enhance the fluorescence of MRFNs still needed further study. It was speculated that the substituted chlorine on CTC might be a good leaving group because of the easy fracture of C–X (Cl).^35–37^ Therefore, CTC could easily form covalent bonds with MRFNs (existing hydroxyl, carboxyl groups, and possibly amino groups) with a high affinity. On the one hand, with an increase in the rigidity of the molecular structure, the energy loss produced by the excited electrons state returning to the ground state was reduced; on the other hand, the self-complexation of MRFNs might be broken, which could reduce the aggregation of MRFNs molecules, so the dispersion state between MRFNs molecules was maintained, thus enhancing the fluorescence of MRFNs.^37–39^ Overall, the clear mechanism of CTC-induced fluorescence enhancement of MRFNs still needs further experiments to prove.

## 4. Conclusions

In summary, the easy-synthesized water-soluble fluorescent probe (MRFNs) was prepared using the “green” method with simple technical processes. Also, the effects of CTC and TC on the fluorescence behaviours of MRFNs in aqueous solutions were studied respectively. The results illustrated that CTC can greatly enhance the fluorescence intensity of MRFNs, while TC can significantly reduce the fluorescence intensity of MRFNs. Furthermore, novel methods for the rapid detection of TC and CTC in aqueous solutions based on the fluorescence behaviours of MRFNs were established, respectively. Eventually, the established methods were successfully adopted for detecting CTC and TC in some environmental water samples. Moreover, the possible mechanisms about each TC on the fluorescence behaviours of MRFNs were also discussed, respectively.

## Conflicts of interest

There are no conflicts to declare.

## Supplementary Material

## References

[cit1] Qiao M., Ying G. G., Singer A. C., Zhu Y. G. (2018). Environ. Int..

[cit2] Pan L., Feng X., Cao M., Zhang S., Huang Y., Xu T., Jing J., Zhang H. (2019). RSC Adv..

[cit3] Sun J., Zeng Q., Tsang D. C. W., Zhu L. Z., Li X. D. (2017). Chemosphere.

[cit4] Lv J., Zhang L., Chen Y., Ye B., Han J., Jin N. (2019). J. Water Health.

[cit5] Yang Y., Song W., Lin H., Wang W., Du L., Xing W. (2018). Environ. Int..

[cit6] Ayoub G., Ghauch A. (2014). Chem. Eng. J..

[cit7] Muscalu A. M., Górecki T. (2018). TrAC, Trends Anal. Chem..

[cit8] Conzuelo F., Gamella M., Campuzano S., Reviejo A. J., Pingarrón J. M. (2012). Anal. Chim. Acta.

[cit9] Wang P., Wu T. H., Zhang Y. (2016). Talanta.

[cit10] Wang B., Zheng Y., Fang D., Kamarianakis Y., Wilson J. R. (2019). Stat. Med..

[cit11] Ben Y., Fu C., Hu M., Liu L., Wong M. H., Zheng C. (2019). Environ. Res..

[cit12] Starzec K., Cristea C., Tertis M., Feier B., Wieczorek M., Koscielniak P., Kochana J. (2020). Bioelectrochemistry.

[cit13] Jadhav M. R., Pudale A., Raut P., Utture S., Ahammed Shabeer T. P., Banerjee K. (2019). Food Chem..

[cit14] Voigt A. M., Skutlarek D., Timm C., Schreiber C., Felder C., Exner M., Faerber H. A. (2020). Environ. Chem..

[cit15] Yang M., Zhang X., Liang Q., Yang B. (2019). Water Res..

[cit16] Wei D., Wu S., Zhu Y. (2017). RSC Adv..

[cit17] Al-Hashimi B., Omer K. M., Rahman H. S. (2020). Arabian J. Chem..

[cit18] Liu H., Ding L., Chen L., Chen Y., Zhou T., Li H., Xu Y., Zhao L., Huang N. (2019). J. Ind. Eng. Chem..

[cit19] Qi H., Teng M., Liu M., Liu S., Li J., Yu H., Teng C., Huang Z., Liu H., Shao Q., Umar A., Ding T., Gao Q., Guo Z. (2019). J. Colloid Interface Sci..

[cit20] Xu Z., Yi X., Wu Q., Zhu Y., Ou M., Xu X. (2016). RSC Adv..

[cit21] Habinshuti I., Chen X., Yu J., Mukeshimana O., Duhoranimana E., Karangwa E., Muhoza B., Zhang M., Xia S., Zhang X. (2019). LWT.

[cit22] Nooshkam M., Varidi M., Verma D. K. (2020). Food Res. Int..

[cit23] Dong J. X., Song X. F., Shi Y., Gao Z. F., Li B. L., Li N. B., Luo H. Q. (2016). Biosens. Bioelectron..

[cit24] Dong J. X., Wang Z. L., Yang Y., Gao Z. F., Li B. L., Jiang H. H., Li N. B., Luo H. Q. (2017). J. Mater. Chem. B.

[cit25] Nasrollahzadeh F., Varidi M., Koocheki A., Hadizadeh F. (2017). Food Res. Int..

[cit26] Xue H., Yan Y., Hou Y., Li G., Hao C. (2018). New J. Chem..

[cit27] Long D., Peng J., Peng H., Xian H., Li S., Wang X., Chen J., Zhang Z., Ni R. (2019). Analyst.

[cit28] Zhang H., Chen H., Pan S., Yang H., Yan J., Hu X. (2018). Luminescence.

[cit29] Zhang Z., Chen J., Duan Y., Liu W., Li D., Yan Z., Yang K. (2018). Luminescence.

[cit30] Anand S. K., Sivasankaran U., Jose A. R., Kumar K. G. (2019). Spectrochim. Acta, Part A.

[cit31] Lin M., Zou H. Y., Yang T., Liu Z. X., Liu H., Huang C. Z. (2016). Nanoscale.

[cit32] Wang Z., Lin J., Gao J., Wang Q. (2016). Mater. Chem. Phys..

[cit33] Yan Y., Liu J. H., Li R. S., Li Y. F., Huang C. Z., Zhen S. J. (2019). Anal. Chim. Acta.

[cit34] Qian S., Qiao L., Xu W., Jiang K., Wang Y., Lin H. (2019). Talanta.

[cit35] Li L., Zhu J.-Y., Hong Y.-J., Li M.-H., Xiao W.-C., Lin M.-J. (2019). Dyes Pigm..

[cit36] Westaway K. C., Fang Y.-r., MacMillar S., Matsson O., Poirier R. A., Islam S. M. (2007). J. Phys. Chem. A.

[cit37] Mandal P., Sahoo D., Sarkar P., Chakraborty K., Das S. (2019). New J. Chem..

[cit38] Chen L., Raohao F., Changchang Z., Xiang L., Changhua Z. (2019). Polym. Degrad. Stab..

[cit39] Yin H. Q., Yin F., Yin X. B. (2019). Chem. Sci..

